# “For us women, flavor is king”: Gender, saf sap and flavor work in urban Senegal

**DOI:** 10.1080/15528014.2023.2268362

**Published:** 2023-12-12

**Authors:** Branwyn Poleykett

**Affiliations:** Department of Anthropology, Faculty of Social and Behavioural Sciences, University of Amsterdam, Amsterdam, Netherlands

**Keywords:** Taste, gender, social reproduction, nutrition, Senegal

## Abstract

Over the past decade home cooking in the Senegalese city of Dakar has come to be dominated by culinary practices of *saf sap*: the incorporation of new commodities and flavor enhancers and the invention of new cooking techniques that intensify the taste of everyday dishes. Producing a well flavored meal is a crucial part of women’s domestic work, but cooks are increasingly critiqued in Dakar, accused of traducing culinary heritage, challenging the authority of elders, and spreading metabolic disease. Drawing on ethnographic research in Senegalese households and qualitative interviews and focus groups with women who prepare food, I introduce the analytical category of “flavor work” to show how the everyday making of taste in a West African city is embedded in historical and contemporary forms of household social reproduction. Controversy over the taste of home cooking reveals how women’s flavor work serves a double reproductive purpose. Flavor work creates culturally coherent and intelligible meals, but it also forms part of broader subsistence strategies: techniques for navigating scarcity and rupture.

## Introduction

In early 2023 I sat down to cook a dish of rice and chicken [*cebuginaar*] with my friend Fatou in the Dakar suburb of Pikine. In the context of an inflationary crisis and very high food prices, this dish would have been too expensive for many people in Pikine, but Fatou’s family had preserved their habit of eating *cebuginaar* together on Sunday afternoons. As it was Fatou’s *aye*, or turn to cook for the whole family, a responsibility that she shared with her sisters in law, she was charged with preparing a meal for around twenty people. Fatou’s husband worked outside of Dakar and had already transferred to her the money that allowed her to perform this task. Once prepared, the meal would be separated into two large bowls, one for women and one for men, and the family would assemble around the bowls to eat together. Because Fatou worked in Dakar during the week she hired a maid to do her domestic work and therefore only cooked herself when her “turn” fell on a weekend. Because this choice sometimes attracted the critique of other family members, Fatou was under pressure to create flavorful and memorable meals, and she was aware that her cooking was closely scrutinized. The moment when the food was presented [Wolof, *taje*] and the smells emanating from the steaming rice revealed the rich flavor of the dish was a moment of pride, but also of anxiety. Would the flavor of the meal be satisfying and recognizable, while also disclosing her personal skill and her *loxo* [hand], the distinctive and idiosyncratic choices she had made in flavoring the meal?

In Senegal home cooks navigate several everyday challenges. They must transform the *dépense quotidienne* or the “daily allowance” provided by their male relatives into adequate meals for large households, while finding workarounds for fluctuating prices or ruptures in availability by substituting ingredients or innovating recipes. In executing this challenging task, cooks must also fulfil their social role as custodians of cultural heritage, reproducing a tight repertoire of national dishes. Women undertake to maintain culinary traditions and to pass on contact with traditional ingredients and dishes to their children. The stakes of performing these complex tasks “successfully” are high. The creation of flavorsome dishes is central to the practice of *mokk pooj*, the ensemble of erotic, emotional and culinary activities that women perform to anticipate and satisfy their husband’s requirements in the social institution of marriage. *Mokk pooj* which roughly translates to the “art of the thigh” (Mustafa 2006), refers broadly to maintaining harmonious domestic relations through satisfying male desires, as well as to the finely ground spices and condiments that flavor a dish (Gilbert 2019). The way that women craft good tasting meals, however, is not just a private affair between women and their partners. Women’s cooking is understood as an extension and expression of their selves; through cooking, women communicate their respect for and adherence to idealized norms of feminine modesty and respect. Women’s cooking brings people together around a pleasurable dish that materializes values of harmony and symbolizes the integrity and complementarity of relationships between men and women and between generations.

Fatou pounded chilies, garlic and onions into a paste to form the base of the dish. To this paste, we added quantities of salt and pepper to build the savory flavor profile that would suffuse the rice. When it came time to add the flavor enhancing stock cubes, however, Fatou turned her back, placing herself between me and the mortar, joking that she would not let me see how many stock cubes she was adding to the dish in case I disapproved of her cooking. As she joked about “hiding” the use of processed foods and industrial flavor enhancers in her cooking, Fatou claimed to be concerned about appearing in my writing as an advocate or practitioner of a ubiquitous but increasingly stigmatized practice: *saf sap. Saf*, often accompanied by the superlative *sap*, literally means delicious, but the word *saf* also identifies a modern style of preparing and flavoring food. *Saf* cooking is based on the combination of stock cubes, oil, mustard, vinegar, salt, sugar, dried fish, smoked fish, fresh parsley and lime, with industrial or “chemical” flavors. In combination, these elements produce a pungent, emphatic and highly savory flavor, the first spoonful or handful of a *saf* dish constricts the throat and floods the taster’s mouth with a rush of saliva, eliciting the exclamation *saf!* Delicious!

I understood Fatou’s concern about being depicted as using “too many” flavor enhancers. Over the past five years *saf sap* has become a subject of significant public concern in Dakar (Ka and Leport 2023; Vialard et al. 2017). In the context of rising rates of diet related chronic disease, nutrition and public health interventions have targeted their public awareness campaigns and interventions not just the nutritional content of meals but the taste of domestic cooking. Flavorsome food is increasingly seen as overstepping boundaries or transgressing limits. Rather than being praised for the delicious flavors they create, young women who traffic in high flavor can be accused of cooking in ways that are destructive of culinary heritage, kinship and social relations, and the health and wellbeing of elders. As women’s flavor work at home spills out into the public realm, women are challenged to remake their practices, and in turn to rebuild the core values that shape cooking and eating, for example by rediscovering and revaluing traditional ingredients, or by cooking simpler and “blander” [Wolof, *lëwët*] dishes. But the challenge of diminishing flavor is a painful one for Senegalese women to rise to, as one woman present at a public health intervention on healthy eating reflected gloomily: “we women are strange, for us, flavor is king.”

In joking about her use of stock cubes, Fatou drew on interventions she had heard on the radio and seen on television encouraging women to diminish the use of salt and stock cubes in their dishes. The “excessive” use of flavor enhancers had been folded into a lively popular discourse about “bad eating” [Wolof, *lekkin bu bon*], supposedly dangerous consumption patterns and practices that had triggered a crisis of debility and a frightening epidemic of sudden death from heart attacks, strokes, or complications of diabetes (Poleykett 2023). Discourses of bad eating, bad food, and bad cooking are fueled by a well-founded perception that West African consumers are unprotected by their governments and routinely receive the lowest quality products that the market has to offer (Ham 2017). The problematization of *saf sap*, however, does not just concern the impact of food on the body, and highly flavored food can endanger more than bodily health and wellbeing. Debates about *saf* cooking within households brought into focus relations between young women and older women, between eating traditions and urban modernity, and between men and women. The “problem” of “bad eating,” in other words, rebounded onto Dakar home cooks, who now had an additional burden to manage as they provisioned their households and prepared food: making food that was not only tasty but “tasteful,” food that, in my interlocuters’ terms, did not “exceed limits” in the pursuit of flavor.

## Ecologies of taste in the kitchen

Writing on taste in the social sciences has long shown how the sensory is a critical part of the social (D. E. D. Sutton 2010). The taste of food shapes collective memories (Holtzman 2009), creates new forms of social stratification and hierarchy (Bonnecase 2016; Bourdieu 1984), and forms (and breaks) relations between cooks and eaters (Stoller and Olkes 1986). In this theoretical tradition, taste is understood as a kind of “sensory epistemology” (Abarca 2006); a matrix for navigating the world and grasping, analyzing and reshaping its values and its material organization. In addition to these rich investigations of the symbolic and representational power of taste to condense meaning, create eaters, and remake worlds, food scholars have analyzed taste across scales, as a sociopolitical phenomenon, a powerful force capable of reshaping landscapes of food production and driving forward radical changes in dietary patterns in practice (Chester 2009; Mintz 1986). These two traditions converge on the study of the incorporation and embrace of new food commodities on global “peripheries.” As the taste of food changes and evolves in response to the availability of new food commodities, taste is an idiom through which modernity can be encountered as potentially pleasurable or full of possibility, but also charged with poignancy and fear of cultural loss and erasure. Instant noodles, stock cubes, and powdered milk appear as “icons” of the evils of the global twenty first century diet, consummate “proletarian hunger killers” in Sidney Mintz’s words, “greas[ing] the wheels of global capitalism” by normalizing and validating a world of commodities through intimate acts of everyday food preparation and consumption (Errington, Fujikura, and Gewertz 2012).

What is missing in this critical literature on the pernicious colonization of traditional diets by global food is an understanding of how modern food commodities meets the reproductive work of households and home cooks. In this article I show that *saf sap* is not just about stock cubes inexorably conquering the communal bowl. Rather, *saf sap* entwines with women’s longstanding reproductive “flavor work,” a set of strategies through which women navigate food access, creating solutions to urban food insecurity. By seeing the production of taste as woven into women’s everyday domestic obligations, I enter into dialogue with and extend theoretical accounts of taste rooted in political ecology paradigms, approaches that aim to understand taste as a material phenomenon located at the intersection of domestic work, embodiment, sensory experience, historical and economic change, and access to food (Hayes-Conroy and Hayes-Conroy 2015; Kinkaid 2019; Nichols 2022; Nisbett 2019). Ecological approaches to eating trace encounters between macrohistorical forces and metabolizing bodies, analyzing how food production, trade agreements, and food policies “guide” commodities into bodies (Hatch, Sternlieb, and Gordon 2019). I argue that work in this emerging tradition has often neglected the role of cooking as a critical mode of interpreting, resisting, querying and reproducing eating. Using a feminist and social reproduction lens to analyze the emergence of *saf sap* as part of women’s navigation of chronic food insecurity, I argue that the critique of women’s flavor work as unhealthy and disruptive of cultural continuity and social relations can be understood as a form of “gendered food injustice” (Brady et al. 2018). Specifically, the new public health focus on food’s flavor in Senegal obscures the ways that women use flavor and mobilize their flavor work to craft situated solutions to domestic pressures, cultural loss, and chronic and persistent urban food insecurity.

Drawing on long term ethnographic research in households in the Dakar suburb of Pikine, I begin by showing how encounters with *saf* tastes have “produced” (Ham 2017) novel kinds of eaters. Women’s flavor work is not just necessary for crafting and maintaining gendered selves, encounters with the flavor of household food can bring into being people with a distinctive taste for “blandness,” or people managing metabolic disease who experience pain and “bitterness” as they eat highly flavored food. I then elaborate further on the conundrum faced by home cooks like Fatou: how to balance the imperative of creating delicious flavors with the demands of other eating repertoires and newer cultural values of simplicity, health, and cultural integrity? Here I show that women draw on narratives of continuity that justify and dignify cooking practices that are increasingly stigmatized. The significance of *saf sap*, however, is not merely symbolic. Flavor work can disrupt relations by materially controlling how food is distributed within the household and who can safely and comfortably eat. I argue that flavor work plays a critical role in social reproduction in Dakar households. I support this argument in the second half of the paper by arguing that as a situated solution to scarcity, flavor work qualifies as life sustaining: making sustenance and satiation possible. Further, flavor work constitutes a daily and critical engagement with the changing materiality of food, and, as I show toward the end of article via a striking ethnographic example, everyday flavor work exists in a close interpretative relationship to changing food production and the vagaries of food access.

## Methodology

This article emerges out of a larger project which examined how people diagnosed with chronic conditions in Dakar developed self-care strategies and tried to adopt new dietary practices. I draw on four months of participant observation in two households in Pikine where I shopped with women and participated in the preparation of midday meals. I also base my analysis on focus group and interview data. With a co-facilitator, Aminata Diallo, we conducted six focus groups in Wolof, each composed of 6–8 women of different ages, who were asked to talk about preparing and eating food in their households. We also carried out follow-up in-depth biographical interviews talking to older women about their experiences of cooking and eating across their lifetimes. The ethnographic data helped to provide a fuller and realistic picture of day-to-day consumption than could be accessed using interview methods. This data revealed the economic difficulties under which home cooks operate, and the strategies they develop to work around those constraints. Detailed fieldnotes were taken throughout the ethnographic observation and coded for emerging perspectives. The focus group data showed how passionately women were interested in *saf sap* and how they analyzed the phenomenon and contested stigmatizing representations of *saf* cooking. Because the focus groups were mixed in age, they also revealed significant conflict between younger and older women over changing norms of cooking. The follow up in-depth biographical interviews provided historical context and time-depth, developing a more detailed picture of the historical context and contested origins of a practice often framed as novel and quintessentially modern.

The criteria for inclusion in the research was that people understood themselves as directly involved in food preparation. This included older women who participated in cooking in a supervisory role, as well as the younger women, their daughters and daughters in law who shopped for and prepared the household meals. While women often reported that they created highly flavored dishes as part of a broad repertoire of obligations they owed their husbands and their male kin, talking to men about taste and about everyday eating was challenging. The deep, varied and sophisticated vocabulary that women used to describe and differentiate tastes was lacking in men’s accounts and male members of the household often claimed near total ignorance about how food was cooked. By focusing only on women’s experience it is difficult for me to evaluate and validate some of the claims made by women in this research, for example, that *saf sap* allows them to present palatable versions of traditional food that would otherwise risk rejection, devaluation and loss. However, I include and analyze these accounts as part of women’s attempts to construct and occupy appropriate gendered identities.

Two social identities shaped my research: my identity as a cook and my identity as an eater and temporary member of the eating collective. As a cook I asked to learn how to create *saf* tastes, a request that I, and others, took very seriously. In Senegal women learn how to cook from their mothers, and “recipes” and techniques are more like “gendered historical transcripts” (McCann 2009) than a generic set of instructions that can be easily shared. Although I certainly tried to acquire some skills, I cannot in sense be considered an initiate of *saf* cooking. I did not acquire much beyond a very basic competence and most tasks were quickly taken off my hands when I slowed down the pace of preparation. As we cooked I asked about *saf sap* and about *saf* technique, asking to know how the delicious and striking flavors of the dish were produced. This was a delicate line of questioning. Aware of the critical attention that *saf sap* received, women were understandably reticent in sharing their culinary practices with an outsider. I rarely saw the full array of *saf sap* technique deployed, and many of the more lurid or extreme techniques described in the following section circulated as urban myths rather than practices that I directly witnessed. I choose to include these stories about *saf sap*, however, in the analysis, as they formed an important part of the problematization and rejection of *saf sap* and were drawn upon to evidence how flavor had gone “too far.”

The second identity that shaped my presence in the field was my position as an eater. Here too my positionality was ambiguous. While I truthfully said when asked that I had come to love the deep and funky tastes of *saf* cooking, I was really happiest during Ramadan when I formed a small eating collective with the people who were not fasting: young children, pregnant women, diabetics and the elderly, people who relied upon or valued “simple” food such as a plate of *mbaxalu saluum*, a “wet” [Wolof, *tooy*] congee-like rice dish flavored with citrus. While I studied changing attitudes to *saf sap* I was aware that it was often my presence as a guest that encouraged people to prepare the “heavy” national dishes that served as the vehicles for experimental and flavorsome cooking. I took what Hannah Garth calls an “embodied approach” to studying everyday eating (Garth 2022), allowing my own body to register its own responses to *saf* cooking. In this way I came to recognize the physical responses to food that women described. I recognized some of the symptoms of a kind of “syndrome” associated with eating *saf* food: overpowering thirst, tightness or constriction in my chest, dizziness, nausea, and sudden sharp headaches. By the end of my fieldwork, in other words, I had no issue “believing” the claim made by older women in interviews and focus groups, that contact with *saf* food could produce challenging effects on the body.

## Making *saf* tastes

Flavor work has long been at the heart of the economic life of Senegalese households, closely related to the gendered division of household labor. In nineteenth century Wolof households women created economic value as well as personal distinction by cultivating spice and pepper crops for domestic use and for sale (Buggenhagen 2012). Oral history interviews with people with rural backgrounds revealed memories of gathering herbs and plants to flavor meals, an activity that tied the production of taste to the specificities of ecology and place (Ka 2019). Knowing how to grow, collect and prepare the plants and animals that make up the traditional flavors of Senegalese food, marine life like the pungent sea snail *yéet*, or the fermented locust bean *nététou*, is part of women’s intimate knowledge (Fall 2002). Flavor work is also a crucial axis of Senegalese women’s economic lives, crafting possibilities for both the sustenance of and reproduction of the household. In the city, for example, skilled cooks can market their abilities by selling their food to their neighbors, or by setting up small restaurants. The work of flavor is a form of what Laura Ann Twagira calls “embodied engineering,” the “complex interplay of skill, knowledge, social meaning, leisure and survival” that makes up the “mundane yet extraordinary” work of women’s daily food production (Twagira 2021, 3). Flavor work is tied to women’s power, both the power of creating value for the household, and the often hidden or disavowed power to create and flavor food exercised by the women who provision and cook day to day in urban households

In the context of the modern city the incorporation of industrial flavor enhancers into West African cuisine appears to offer a convenient solution to the laborious task of building flavor out of ingredients that are sensorially complex and challenging, as well as sometimes scarce. A young woman cooking in Dakar can choose between a vast range of powdered products that reproduce the umami flavor of smoked fish and locust bean paste. A reliable, shelf stable, ubiquitously available stock cube that can be purchased cheaply seems to extend the promise of food security and plentiful and predictable urban eating. Where there is nutritional loss, for the products that replace traditional flavor bringing ingredients have no nutritional value, there might be significant gains, both in convenience and also in tasting pleasure. Indeed, as I explore below, before *saf sap* came to be questioned as culturally inappropriate and unhealthy I found that people often praised the sensory experience of “clean” *saf* flavors of stock cubes and soy sauce over the more complex tastes of traditional food.

Anthropologists have extensively studied the incorporation of new commodities into local foodways and traditional diets, examining strategies of “vernacularisation” as new food commodities are folded into local cooking and adapted and reshaped to local palates and desires (Simpson Miller 2021; Caldwell 2004; Baviskar 2018). Writing on dietary change and the integration of new commodities into diets in Belize, Richard Wilk suggested a number of typologies for the “creolization” of cuisine (Wilk 2006). Creolization can involve processes of “blending,” “submersion,” “substitution” and “compression,” all techniques for integrating new foods in such a way that they become indigenized and recognized as an integral and indispensable part of local eating. The widespread adoption of practices of *saf sap* in Dakar resemble in some ways the trajectories of dietary change that Wilk describes. However, the history and emergence of *saf sap* has some key specificities, and I outline these particularities in this section. I have already shown that *saf sap* is problematized because it *replaces* nutritionally valuable and traditional tastes with ersatz flavor enhancers. A straightforward substitution narrative, however, does not cover what is taken to be “wrong” with *saf sap*, or why the practice might be seen as subversive of culinary culture. In this section I describe why *saf* cooks might be so closely scrutinized and criticized. I explore the intensification thesis and the non-respect for “limits,” the use of “tricks” [*astuces*] or shortcuts, and the secretive incorporation of non-foods into the collective bowl.

In Senegalese households the iconic national dishes are eaten in the afternoon. This meal is a powerful moment of shared pleasure and togetherness and the midday meal is considered the most significant vehicle for the cook’s singular skills with flavor. The midday dishes are categorized according to how many pots they are prepared in: *benn cinn* (one pot), or *ñaari cin* (two pots). For *ceebu jen*, the Senegalese national dish, an intensely glamorous and complex meal deeply associated with urban eating, rice is simmered for two to three hours as fish and vegetables poach in the deeply flavored bouillon and infuse their flavor. For *mafe*, a “two pot” dish, chicken is heaped onto a highly flavored peanut sauce which is then served over plain boiled rice, while each eater around the bowl selectively samples fresh chili paste and lime according to their personal taste. Flavor in Senegalese cooking is the outcome of a base of pounded onion, garlic, parsley and fermented pastes, the interplay of deep flavors of smoked and fresh fish, and the fresh citrus additions to the bowl of leaves of hibiscus and sorrel. Flavor is also produced through the cook’s technique, in particular the manipulation of heat and time that allows the flavor to suffuse the rice.

Aminata Sow Fall in her book about Senegalese cooking describes the critical role of flavor in mediating between layers of the dish. Cooks use flavor to create differentiation and depth within the shared rice bowl. Flavor ingredients, for example, might “bury” or hide and then disclose a new item, piquing the interest and appetite of eaters as they move through the communal bowl (Fall 2002). Older women told me that modern *saf* techniques had abandoned this subtle and skilled use of flavor. Younger women, they often complained, exploited new ingredients to cook fast, boiling the food on a high gas flame, and added tastes at the end, sometimes sprinkling powdered flavor enhancers directly on the dish. Rather than working with and through all of the other elements of the dish, many modern *saf* techniques take place on top of the “natural” flavors that emerge during cooking. This seemed to result in a monotonous, undifferentiated high flavor throughout the dish, and not the distributed, uneven and surprising revelation of flavors that characterized the traditional and skillful assemblage of layers of taste cooked slowly over charcoal.

The critique of *saf sap*, then, goes beyond the simple substitution of traditional sources of flavor with ersatz modern products. *Saf* cooks also stand accused of going beyond the boundaries and limits of natural flavors by overloading the bowl with an excessive amount of flavor enhancement. As one Senegalese anthropologist told me, it is not the practice of safle, or making delicious which is intrinsically problematic, for Senegalese have always valued delicious and savory food, but the *“sap,”* the superlative, that implies an excessive pursuit of flavor. Young women were often discussed as being in competition with one another in a kind of race to the bottom to create excessive, delicious, but also puzzling and unplaceable tastes. It is true that young women created and shared “tricks” [French, *astuces*], approaches to cooking that were not recognizable to their older relatives. As David Sutton shows in his discussion of “tricks” in Greek cooking, a “trick” is a kind of portable solution: a “unit” of embodied knowledge that allows cooks to “deal with certain contingencies, to make the most of new or old ingredients, or to produce familiar and unfamiliar flavours” (D. Sutton 2018, 89). The tricks particularly associated with the denigration of *saf sap* were pieces of technique that involved the incorporation of non-foods into the dish to simulate tastes that people struggled to achieve naturally. For example, women were said to add powdered Nescafe coffee to their dishes of *ceebu jen* to add the distinctive “golden” tint to the rice that bespoke skilled cooking. Women also added heaped spoonfuls of ash from the incense pot to give a “braised” taste to meat dishes, a taste that was hard to achieve when cooking “fast” over precious and costly gas. Another “trick” that I did not see directly but that was very criticized was mashing cosmetics and shea butter into ceremonial meals to create a high sheen and finish to the dish.

I have shown how *saf sap* is seen to undermine and traduce culinary tradition by pushing flavor past the “limits” that are deemed recognizable and acceptable. While people are certainly concerned with the overuse of stock cubes and flavor enhancers, the critique of *saf sap* and the stigmatization of the young women who innovate domestic cooking is also to do with speed, new techniques that are seen as shortcuts, and young women’s use of novel ingredients to simulate skill. Driven by the young women who cook day to day for their families in urban households, ongoing experiments with taste test the boundaries of acceptable consumption. As young women create new tastes and techniques, they challenge values of propriety and restraint, as well as newer repertoires of “healthy eating,” and in doing so they come into direct conflict with their relatives.

## “It is hard to feel bitterness towards your child”: kinship and flavor

While conducting interviews about changing cooking techniques in the Dakar suburb of Pikine I met with a woman in her seventies called Khady. As our conversation progressed, it became clear that speaking about cooking and eating was for Khady a significant source of tension and shame. Although Khady was one of the eldest members of her large household, a respected person who should have enjoyed the privilege of eating from the communal bowl without restriction [Wolof, *lekk ba sur*], she admitted that in her household she had difficulty eating. The reason for this was her young relatives who prepared the food. As she said, “young people these days only know how to cook *saf*” [Wolof, *xale noom dañuy deflu saf rek*]. Khady recalled the food of her youth as very different. She remembered it as “light” [Wolof, *ouyof*] compared to the “dry” and “heavy” dishes that now dominated in her household. When she was younger, Khady explained, cooks “respected the limits,” today the pursuit of flavor at all costs has taken over, and Khady described herself as powerless to prevent her young kin from overloading the communal bowl with *saf* paraphernalia. She claimed that she stood over them, cajoling, instructing, imploring them to make “plain” food that older members of the household could tolerate, but as soon as her back was turned they would inevitably add stock cubes, sugar, salt, baggies of what were often referred to as “Indian spices” and chemical products [French, *produits chimiques*] of unknown origin.

For Khady, modern food tasted not like cleanliness, propriety, restraint, cosmopolitanism, or any of the many values that might be embodied by Senegalese food, but the bitterness [Wolof, *naqar*] of her diminished status. When she tasted another over-flavored dish of *saf* food, she described the emotion that she struggled with, saying “it is hard to feel bitterness towards your child.” The bitterness that Khady felt came from a sense that her nutritional needs were not taken seriously, and her experience illustrates the way that *saf sap* can be used to select or control who could eat collective food. As Khady approached her relatives *saf* food cautiously, limiting herself to a small amount, the flavor of food had a significant impact on how household food resources were distributed, and which recipients of those resources were favored and might be able to expect to eat well. Khady’s “bitterness” is a bitterness borne of hunger and restriction, and the anger at receiving what she deemed disrespectful of inadequate care in her old age. The cacophony of domestic flavor became a prism through which people discussed their precarious interdependence and the limitations of their relations with others.

For older people, *saf* cooking, could, in extreme cases, be interpreted as abandonment. While it was difficult to get younger women to speak directly to the quality of the nutritional care they provided for their elders, it was clear that their flavor work was a practice through which they imagined or materially negotiated space from their relatives and their domestic obligations. Imagining autonomy from onerous domestic obligations increasingly became articulated as a desire for “bland food.” My friend Mariama, for example, told me: “I just want to cook for me and my child. If I were only cooking for my son I would carefully prepare for him simple and plain meals, flavor with hibiscus and tamarind, not with chemicals.” As Mariama’s wishful tone here implies, few people were able to negotiate independence from the families within which they were obliged to cook, and few people actually produced the “bland” meals they claimed to wish to eat. Indeed, in reality, as I described in the opening vignette, producing a bland meal would have been the source of significant shame. And yet “blandness” as an abstract desire, floating free from the messy reality of complex everyday eating, became increasingly prized. People could mobilize a taste for bland food as a marker of cultural distinction and a sign of their appreciation for a modern sensorium, to signal that they were receptive to modern and medical norms of eating. They might also, as Mariama dreamed of doing, use a taste for bland food to justify morally risky actions they dreamed of taking, to establish distance and autonomy from the eating collectives in which they were embedded and to which they were morally obligated to contribute their money and their work.

So far I have opened up some of the practical ways that women’s flavor work makes and breaks relations in households. Flavor work is paradoxical, it is both an onerous obligation, a potential source of shame and spoiled identities, *and* a way of subtly remaking, or at least questioning, the composition of the household and the eating collective. Rather than “only” using flavor in the socially acceptable sense, to embody their feminine identities and demonstrate respect for their kin, young women stand accused of mobilizing flavor work to restrict or enable access to food. While this might be interpreted as a violation of norms and obligations of kinship, I have suggested that using flavor as a path to different kinds of agentive self-expression, economic participation and independence from kin is consistent with how women have historically worked with flavor in Senegalese households to craft meaning and make opportunity. Khady’s story, and the “bitterness” that she experienced in tasting the highly flavored food prepared by her relatives, shows that the taste of food triggers conversations and conflicts about kinship and its limits. *Saf sap* also illuminates the agency of the young women cooks who, in Khady’s home, would not listen to and take account of the preferences of older and more vulnerable eaters, or respect their nutritional needs. Whether imagined or desired, as in Mariama’s case, or materially enacted, by Khady’s granddaughters, *saf sap* provides a set of practices through which young women can experiment with loosening bonds and finessing obligations, extending their agency through new kinds of flavor work.

## Traditional tastes and “kitchen sovereignty”

The “solution” to excessive domestic flavor is often posited as a desired “return” to traditional flavor work, a rediscovery of how people used to eat before industrial flavor enhancers and novel techniques remade the collective bowl, imperiling public health and kinship relations in the process. In this section I examine the relationship of *saf sap* to “traditional” eating and describe how cooks craft arguments in support of *saf* tastes, claiming that *safle* is about maintaining contact with traditional foods, and not erasing them. The moral risk of women’s flavor work, in other words, can be justified by positioning it as a *translation* of traditional eating into a modern, urban register; creating a diet that is sustainable because it appeals to modern, urban palates. Women cooks saw their own practices not as destructive, but as a form of what Anny Gaul, drawing on Lauren Berlant calls “kitchen sovereignty,” a form of “personal or practical sovereignty” characterized by acts of “lateral agency.” Kitchen sovereignty, according to Gaul, can be understood as day to day “modest” practices of food sovereignty embedded in and primarily concerned by the “immediacies of everyday nourishment” (Gaul 2022).

During my fieldwork I had an unusual opportunity to taste the suppressed and supposedly much-desired flavors of the rural past for myself while attending a food sovereignty and development workshop organized by an NGO that promoted restorative cultural practice. I listened to impassioned speeches denouncing *saf* cooking and *saf* cooks and imploring young women to stop cooking rice-based *benn cinn* dishes and instead to ask their grandmothers for forgotten recipes. After the speeches I circulated in the courtyard where women dressed in traditional costumes showcased the cooking of their ethnic group, offering people the chance to taste a dish that exemplified their traditional values and culinary knowledge. People around me expressed their appreciation for the dishes, sharing pieces of their own identity and biography, reminiscing about times they had tried something very much like it in their villages. I was keen to taste these fragments of suppressed culinary practice, hoping that it would allow me, if only in a small and imperfect way, to share in the memories of an era of more simple and unembellished eating that I had heard when I recorded life history interviews with women in Pikine. I chose a dish with a Sereer heritage, a plate of *ngurbaan*, in honor of Fatou, an urbanized Sereer woman (although when I later told Fatou that I had eaten *ngurbaan* she laughed, claiming to have never tasted it, and saying it was such an old fashioned and outmoded dish that she was not sure that it was even any longer prepared in villages).

The *ngurbaan* was a dish of *sankhal* or millet-based *cous cous Sénégalais* flavored with hibiscus, groundnut and dried fish. I was familiar with the ingredients. As I often observed in urban households, the basic elements of traditional dishes were often “flipped,” turning them into a sweet dish based on sugar, yogurt and condensed milk. Indeed, transforming “tough” traditional dishes that are considered sensorially challenging, from savory to sweet constitutes part of the apparatus of contemporary *saf* cooking. The *ngurbaan* I tasted at the workshop, however, was not like the sugary comfort food I ate with pleasure in Pikine. Without the sparkling, “chemical” topnote of *saf*, or the sweetness of sugar and milk, the taste of the peanut was overwhelming, and I found the “wet” textures, in contrast to modern “dry” cooking, unpleasantly and mouth-coatingly dense. Whereas in Fatou’s house in Pikine practices of *saf sap* tended to balance out the strong flavors of dried fish, the traditional dish was, to my taste, dominated by its strident flavor. Tasting “traditional” food without *saf* artistry powerfully brought home to me a point that Fatou often made as we cooked: *saf* “tricks” are about prolonging and maintaining peoples’ contact with traditional ingredients and techniques, mollifying modern palates, not destroying culinary heritage.

Young women in Dakar who are critiqued for innovating home cooking defend *saf sap* through references to tradition and heritage. Women see everyday acts of creative, lateral translation: “flipping” traditional millet-based dishes from savory to sweet; or balancing out traditional flavor profiles with modern additions, as a critical part of their flavor work, their work of fulfilling the multiple, even contradictory, expectations and obligations that weigh on cooks. *Saf* cooks seek to position themselves as the social custodians of tradition, by making culinary heritage something that does not have to be prepared on the margins of urban life, or carefully curated as a “taste of the past,” but as a living and evolving tradition underpinning everyday household eating. Here I have considered how the official practice of food sovereignty “from above,” represented by the food sovereignty workshop, might construct the desirability of direct contact with traditional food. I have contrasted this with the everyday practice of *saf sap*, making savory dishes sweet, or muddling [*jaxase*] a strong flavor to make it more appealing. Constructed by women cooks as continuity, *saf sap* appears as a powerful example of “kitchen sovereignty,” which, as Anny Gaul argues, can often “look like narratively asserting the historical continuity of a dish and downplaying the ways it has been altered or modernized” (Gaul 2022).

While *saf sap* is rooted in long histories of flavor work, the latest turn in the complex history of *saf sap* risks the reputation and identity of *saf* cooks, whose experiments with domestic flavor are critiqued as the bitterness of elders within the household joins a powerful public health discourse outside of the household about the bodily dangers of highly flavored food. These critiques of everyday cooking, however, obscure the practice and meaning of women’s flavor work, they belie, for example, how women might use desirable flavors to maintain contact with heritage ingredients, by using traditional cereals in sweet foods, rather than in their traditional form as the basis of a savory dish. These kinds of formal experiments, of lateral translation, of flipping dishes from savory to sweet, and of balancing out traditional and nutritious sources of flavor with modern products are as an important a part of the practice of *saf sap* as loading dishes with stock cubes and soy sauce, and one that might be reclaimed as an appropriate experimentation that is respectful of eating traditions.

## *Saf sap* and ecologies of eating

Through an engagement with the concept of “kitchen sovereignty” and an examination of how *saf sap* is the outcome of sedimented “tricks,” or situated experimentations with the material affordances of food, a more nuanced picture is developed of where urban practices of *saf sap* came from and why, despite significant critique of *saf* cooks, they appear to be deeply embedded in urban cooking. Women cooks are often called upon to defend and explain their flavor practices, making reference to ideas of heritage and tradition to justify the way that they prepare food. I now turn to a consideration of the “outside” of *saf sap*, or how women’s flavor work emerges from continual adjustment to and dialogue with material pressures and questions of access and supply. In this way, I argue, women’s flavor work can be understood as a crucial part of urban social reproduction.

In my interviews and encounters with the public health authorities who sought to regulate *saf sap* I often heard that *saf sap* was a thoroughly contemporary invention, no more than fifteen years old. One activist told me for example that she was convinced that there was no *saf sap*, no intense or “out of control” experiments with domestic flavor, before the global food price crisis in 2007–2010. The moment of crisis delivered a sharp shock to urban households, with the price of imported rice in particular rising dramatically. Rather than switching away from rice-based dishes, however, households went into debt to buy rice, and began diminishing the amount of food prepared day to day (Resnick 2014). In other words, under acute pressure, low-income households made a series of seemingly nutritionally “irrational” decisions in the interests of maintaining their valued way of eating, and exuberant flavor was used to disguise scarcity, or mobilized as a way of simulating satiety and stretching limited resources.

Here women’s flavor work appears as under a different guise, not as an expressive or hedonistic practice focused on creating eating pleasure at all costs, but, rather as a coping strategy, or a response to constraint. Following Bourdieu’s argument, *saf* cooks might be seen as working to create a valued aesthetic and sensory repertoire out of what is fundamentally an economic necessity. Stories about *saf sap* can be used to index *both* poverty *and* excess. Like Brad Weiss’s example of the meatball, *saf sap* seems to *simultaneously* “express economizing limit *and/or* excessive celebration in the very same material form” (Weiss 2022).

In an interview that I conducted in Pikine, Aissatou, a woman in her eighties told me a story that captured the dynamics of dietary change over the past fifty years. In Senegal young girls often learn the first principles of balancing and composing dishes by shopping for food. In the 1970s Aissatou said that she sent her young daughters to the market to shop with money tied in four ends of a shawl: one corner of the shawl contained money for rice, one for vegetables, one for fish and one for flavor. In the 1970s and 1980s, Aissatou explained, each corner of the shawl contained roughly the same amount of money. A young woman learning to shop and cook now in Dakar would observe that the relative cost of each part of the dish existed in very different relation to one another, necessitating different kinds of investment in each part of the bowl. In particular, the cost of rice and protein would fluctuate, but remain a demanding burden on households, while the cost of flavor has become over time significantly cheaper.

I am drawn to Aissatou’s story, with its striking visual and material organization, because of what it might be able to tell us about dietary change as it is lived over time, and its positioning of taste as part of a set of material and embodied encounters with the ensemble of the meal. The practical mnemonic for balancing and composing meals vividly illustrates how changes in availability change the composition of the dish, with flavor playing a compensatory role as access to staples and protein falls away temporarily, or steadily diminishes over time. The story of the scarf exemplifies the dramatic impact on households of the incorporation of Senegal into a highly unstable global food system. The cost of food has not only risen, prices of key commodities have fallen out of predictable relation to one another, with the cost of staples and proteins rising against the cheapening of flavor. In the place of “diet,” something an individual might possess and work on, and something an individual might have the capacity to change, the scarf model instead opens up a local theory of dietary change embedded in what anthropologist Sarah Besky calls the “social ecology” of eating (Besky 2014): the dynamic and delicate relationships between ecologies, economies, producers and consumers, relations that make up practices of *saf sap*, just as much as mustard sachets, stock cubes, and fermented fish. The scarf model delicately and discretely indicates, in an idiom enriched by respect for the challenges faced by home cooks, that in the absence of other indicators of adequate eating, flavor can become a staple part of the meal. As flavor becomes as central to the reproduction of eating as sardines, meat and rice, it also becomes the vehicle for novel forms of problematization, many of which rebound onto cooks, threatening to spoil carefully cultivated, gendered identities that anchor Senegalese domestic life.

The scarf story brings us into the heart of the marketplace and returns us to the core dilemmas and challenges examined in the opening vignette. Women undertake vital reproductive work in translating the household’s financial resources into the maximum amount of meaningful and nourishing food. Following Aissatou, an emphasis on social reproduction can show us how flavor practices connect consumers of food to producers in a wider food system, revealing an important “continuity between the crisis of social reproduction of the producers and consumers of food” (Çelik 2023). These continuities are highly visible to women who shop day to day. When women choose to use more flavor to hide the taste of low quality or spoiled vegetables, or when they choose to spend more money on flavor enhancers to simulate satiation when there is less money available in the household or because food is more expensive, women’s flavor work mediates, interprets, and mitigates broader constraints in access to food.

## Conclusion

The case of *saf sap* shows how women translate a new repertoire of ingredients into their cooking and combine these with novel experimental techniques for preparing and flavoring food. I have drawn upon a long tradition in food studies of understanding the creative “creolization” of cuisine as global food systems spread new foods from “centre” to “periphery.” I have suggested that the case of *saf sap* offers a distinctive interpretation and extension of this literature, it allows us to see flavor as a critical part of “life’s work” (Mitchell, Katz, and Marston 2005), a modality of reproducing not just culturally intelligible tastes, but a way of sustaining life. This reproduction takes place both on a culinary and symbolic level, maintaining the cultural integrity and recognizability of cooking, and on a social and economic level, where flavor is part of practices of subsistence and a strategy for navigating scarcity. While the first and primarily cultural meaning of the patterned reproduction of recognized tastes is socially recognized and debated, the second set of *saf sap* practices, social activities that are rooted in constraint and economic precarity, are often silenced, disavowed, or ignored. This obfuscation of the connection of flavor to reproductive work and household subsistence leaves women vulnerable to critique and devaluation. By seeing a stigmatized practice from the perspective of markets, kitchens, and households, *saf sap* emerges as a creative response to food insecurities and challenges. The example of *saf sap* enriches the longstanding study of the incorporation of new food commodities and the seemingly inexorable spread of new foods from “centre” to “periphery.” If women cooks struggle to imagine a world without *saf sap*, it is because that world would be one of far greater security, food justice, and material abundance than the world they currently inhabit.
